# AI‐surrogate structure and dose correlation for left anterior descending artery in lung stereotactic body radiotherapy

**DOI:** 10.1002/acm2.70703

**Published:** 2026-07-08

**Authors:** Grace Chang, Quan Chen, Randa Tao, Libing Zhu, Yi Rong, Nathan Y. Yu

**Affiliations:** ^1^ Department of Radiation Oncology Mayo Clinic Arizona Phoenix Arizona USA

**Keywords:** AI segmentation, left descending artery, Lung SBRT, mean heart dose

## Abstract

**Background:**

The left anterior descending (LAD) coronary artery is a critical organ‐at‐risk in thoracic radiotherapy. In thoracic stereotactic body radiation therapy (SBRT), sharp dose gradients and poor LAD contrast on 4DCT scans introduce uncertainty in LAD dose assessment.

**Methods:**

We evaluated the performance of an AI‐generated LAD region structure as a surrogate for manual LAD contours in early‐stage NSCLC. We used deep inspiration breath hold (DIBH) scans from 49 patients with manually delineated LADs; planning risk volume contours were expanded by 3 mm. Then, 96 lung SBRT dose maps were registered by matching the left anterior portion of the heart contours. Dice similarity coefficient (DSC), inclusion ratios (IR), dose–volume histogram (DVH) metrics, linear regression, and linear mixed models were used to compare the structures. The correlation of the AI LAD region and LAD dose to mean heart dose (MHD) was assessed.

**Results:**

The AI LAD region showed strong correlation with manual LAD contours for maximum dose (D1%, *R* ^2^ = 0.93) and volume receiving ≥10 Gy (V10Gy, *R* ^2^ = 0.81). The planning risk volume also demonstrated high correlation to the AI LAD region. MHD was a poor predictor of LAD dose (*R* ^2^≈0.4). Additional dose metric correlations were assessed.

**Discussion:**

To investigate dose volume effects to the LAD during radiotherapy for stage I NSCLC, a robust segmentation paradigm must be established. It must be easily implementable on typical imaging acquired during treatment, with an emphasis on clinical translatability. Our model has shown that the AI LAD region has a strong correlation with LAD dose under typical lung SBRT doses and supports its use for further investigation into LAD dose volume constraints during lung SBRT.

**Conclusion:**

AI LAD region provides a clinically feasible and scalable alternative to manual LAD contouring for dose estimation in lung SBRT. Its strong correlation with key dose metrics validates its use in large‐scale retrospective and prospective studies.

## 1 INTRODUCTION

The left anterior descending (LAD) coronary artery is a critical organ at risk (OAR) in thoracic radiation therapy, with elevated doses linked to increased risk of major adverse cardiac events (MACE) and mortality.^1^, ^2^, ^3^, ^4^, ^5^ While mean heart dose (MHD) is commonly used as a surrogate for cardiac radiation exposure, it poorly correlates with LAD dose in non‐small cell lung cancer patients (NSCLC) receiving intensity modulated radiation therapy (IMRT) or stereotactic body radiation therapy (SBRT).^6^, ^7^, ^8^ Accurate LAD dose estimation is particularly important in lung SBRT, where steep dose gradients, hypofractionation and tumor proximity to the heart can result in high LAD exposure.^9^, ^10^, ^11^ Additionally, proposed LAD‐specific dose metrics, such as the percentage of the LAD volume receiving 15 Gy (V15Gy[%]), have been independently associated with cardiac toxicity and mortality in large cohorts of patients with locally advanced NSCLC treated with IMRT and 3D conformal radiotherapy (3D‐CRT).^12^ Specifically, McKenzie et al analyzed RTOG 0617 data and predicted a 20% overall survival drop at 2 years with as little as 15 Gy of radiation to 10% or more of the LAD.^2^ Zureick et al. examined 375 breast cancer radiotherapy patients and demonstrated a threshold as low as 3 Gy for average LAD dose can predict poor heart outcomes, such as stents or heart attacks post radiation therapy.^13^ The analogous SBRT DVH metric remains unknown, and further investigations are challenging as routine LAD contouring on 4DCT scans typical of lung SBRT are subject to low contrast and therefore a greater expertise burden.

Several groups have shown acceptable automatic LAD contouring using a range of techniques from deep learning to multi‐atlas‐based approaches limited to patients with breast cancer.^14^, ^15^, ^16^, ^17^ These models are aided by clearer breath hold scans, and similar studies in lung SBRT are challenged by reduced cardiac contrast of typical 4DCT techniques.^18^, ^19^, ^20^ Chin et al demonstrated strong correlation in mean dose and maximum dose between automatic atlas vs manual LAD contours on a limited set of 30 lung cancer patients but only obtained a Dice Similarity Coefficient (DSC) of 0.06, indicating minimal contour similarity.^21^ Similarly, Olloni et al demonstrated strong correlation of mean dose with a larger simulated cohort but had low DSC scores and failed to investigate more clinically relevant dose metrics.^22^ Chen et al implemented an expanded planning risk volume (PRV) structure for the LAD in their deep learning segmentation model, which showed improved Dice scores of ∼0.6 on 100 lung cancer patients, prompting the search for surrogate structures for application to lung cancer.^23^

## 2 MATERIALS AND METHODS

We evaluated the use of an institutional deep learning model in use for breast cancer for the generation of an LAD planning risk volume (LAD_REGION) as a surrogate for manual LAD contours in lung SBRT. By registering ground truth cardiac anatomy from high‐quality DIBH scans with appropriate motion margins to lung SBRT dose maps, we simulated LAD/LAD_REGION dose exposure scenarios. This design allowed assessment of whether the LAD_REGION could serve as a dosimetrically correlated surrogate for a manually contoured LAD in lung SBRT. The robustness of the model on 4DCT scans is under investigation and briefly mentioned in the discussion.

### 2.1 LAD_REGION

**FIGURE 1:**
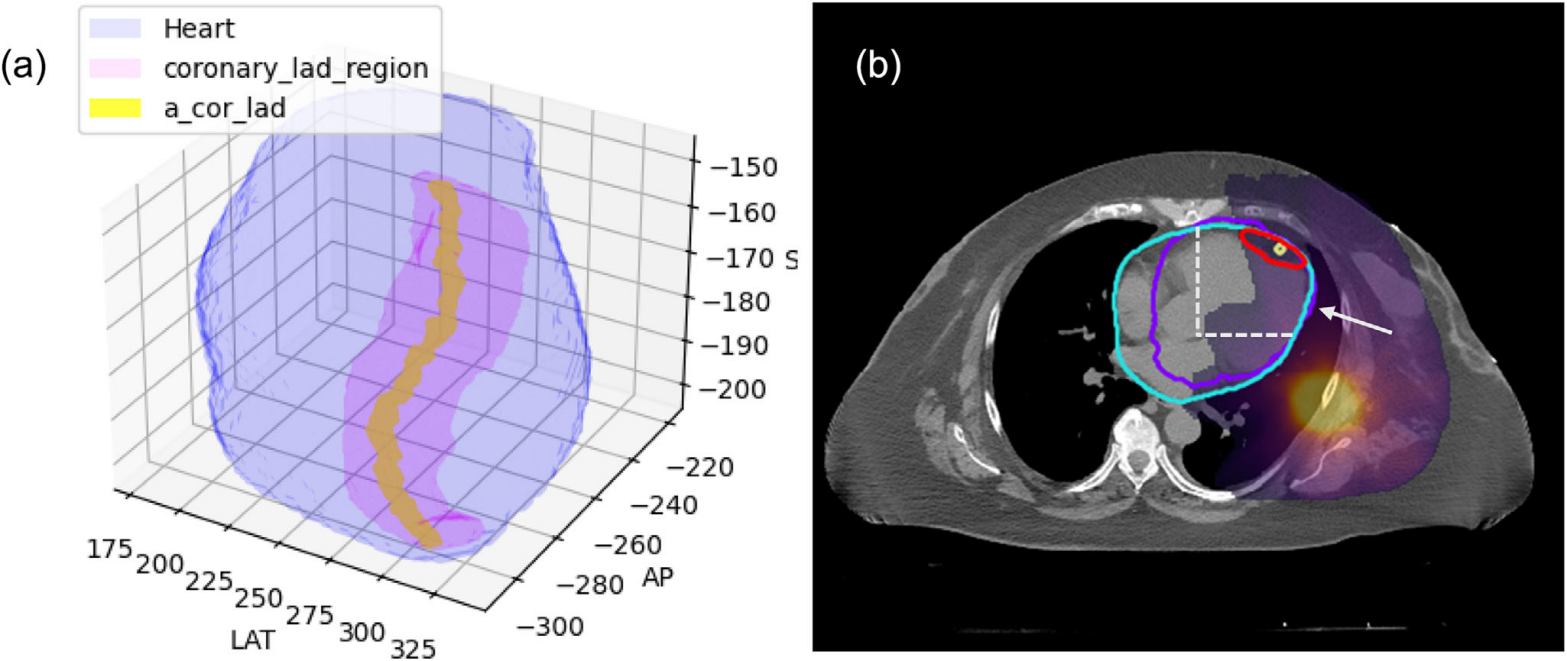
**Region definition and registration approach**. (A) The LAD and LAD_REGION visually displayed for one patient. (B) The dose distribution and purple heart contour are associated with a lung SBRT patient, whereas the displayed CT, blue heart contour, red LAD_REGION and yellow LAD contours are associated with a breast cancer patient. The registration is focused axially on the left anterior portion of the heart as indicated by the white dashed lines and arrow to preserve the dosimetric impact of the heart lung interface.

LAD_REGION is segmented by the proprietary clinical deep learning model created from a collaboration between our institution and Google DeepMind (Figure 1). The auto segmentation model is based on the 32‐slice window 3D U‐Net architecture following the training guidelines set by a previous head and neck model.^24^ Training data began as a pool of 400 weakly curated patients, which were carefully curated to a representative set of 50 patients. A separate group of 30 expert gold standard contour sets was used for validation. This new thoracic model has been validated and deployed at our institution as of 2024 for breast treatments. The LAD_REGION is briefly defined as extending craniocaudally from the left main coronary artery to the apex of the heart, running along the interventricular groove, and incorporating adjacent pericardium. The group proposing this definition of LAD planning risk volume demonstrated it reliably includes the LAD with an inclusion ratio (IR) of 96%, defined by the percentage of the LAD structure within the region structure.^25^

### 2.2 Data curation

Clinical guidelines standardized the contouring of left breast DIBH patients. Five trained dosimetrists generated the manual LAD based on a unified cardiac atlas, using a 4 mm diameter brush.^26^ We retrospectively analyzed a cohort of 49 left‐sided breast cancer patients who previously underwent 3D‐CRT with deep inspiration breath hold (DIBH) techniques, for whom high‐quality, manually delineated LAD contours and auto segmented LAD_REGION contours were readily available and extracted from the clinical structure set. LAD_PRV_3 mm was derived to mimic a standard PRV in lung cancer, created by isotropic expansion of the LAD by 3 mm using the SimpleITK Python library. A representative set of 96 lung SBRT patients was then identified; all receiving 50 Gy in 5 fractions to lung lesions. Their structure sets, CTs, and dose DICOM files were used for analysis.

### 2.3 Simulation approach

Validation and implementation of this model require two stages. First, it requires validation that the LAD_REGION as contoured by the model dosimetrically correlates to the LAD in high dose gradients typical of lung SBRT. This is preferably performed on DIBH scans, as this is the model's validated regime. DIBH scans also allow more robust LAD contouring due to increased contrast. Additionally, simulated dose mapping was necessary to evaluate LAD dose across a large cohort while preserving anatomical consistency, given the limited availability of robust LAD contours on typical average 4DCT lung SBRT scans. Once this benchmarking is completed and the correlation between LAD_REGION and the LAD confirmed, we can proceed to test the model robustness on 4DCT scans.

Each contour set from the DIBH scans was rigidly registered onto the 96 lung SBRT dose maps using SimpleITK in Python, with the heart serving as the matching structure in the registration. To preserve the inhomogeneity effects within the sharp electron density change between the heart and lung, the left anterior portion of the heart contour served as a matching point in the axial plane, whereas the center of mass was used to register the images in the craniocaudal direction (Figure 1). This approach simulated 4704 different geometric configurations of lung tumor and LAD/LAD_REGION typical of stage I NSCLC, while preserving the high‐quality ground truth LAD contours on DIBH scans.

To evaluate the utility of the LAD_REGION as a surrogate for the LAD in dosimetric analysis, we employed several validation strategies. While DSC is often used for contour validation, the significant volumetric disparity and different anatomical definition between the LAD_REGION and LAD rendered it unsuitable. DSC was calculated and is mentioned in the discussion for consistency with the literature, though we focus on comparing dose‐volume histogram (DVH) metrics between the two structures.

### 2.4 Statistical analyzes

To assess dosimetric correlation, linear regression (LAD_REGION vs LAD) was done for D1%– D50%, and V5Gy–V50Gy using the standard Python statistics library. The coefficient of determination (*R* ^2^) indicates the quality of the fit. Data points that resulted in no dose (under 2 cGy) to the LAD or LAD_REGION were omitted from the fits to minimize low dose bias. This cut off dose was progressively increased up to 20 Gy to assess the strength of the correlation at clinically relevant LAD doses. Histograms of residuals were generated using SciPy and Matplotlib to assess residual normality to confirm the appropriate application of linear regression. Bland Altman plots were also generated.

While the in‐silico design improves the size of our sample, it is necessary to understand potential bias in the form of clustering. In addition to creating interactive plots to visually check for clustering, we fit a linear mixed model created in Python using the package statsmodels. Structure type (LAD_REGION vs. LAD) was included as a fixed effect, and contoured patient ID as a random effect. For each dose‐volume statistic analyzed, the variance attributed to the random effect indicates the influence of patient‐specific contouring. A low group‐level variance suggests that contoured patient ID did not significantly contribute to clustering in the regression model. This implies that the anatomical selection process did not introduce substantial bias and that the sample is appropriately representative for the intended analysis.

Additional validation included repeating the linear regression with LAD_PRV_3 mm and the LAD_REGION. The 3 mm radial expansion of the LAD results in a ∼ 1 cm diameter structure, which models suggested cardiorespiratory motion margins and mimics a true LAD planning risk volume contour.^27^, ^28^ As a final validation step, we examined the correlation between MHD and LAD V10Gy[%] in our simulated dataset as a sanity check to reproduce the known pattern of poor correlation.^6^

## 3 RESULTS

### 3.1 Inclusion ratios (IR)

We calculated an average IR of 99% (standard deviation of 0.03%) for the LAD within the LAD_REGION. A lower average IR of 78% (standard deviation of 0.07%) was seen for the LAD_PRV_3 mm within the LAD_REGION, mostly due to the anterior expansion of the isotropic PRV not present in the LAD_REGION. A distribution of IR values is available in Figure S1.

### 3.2 Dosimetric correlations

**FIGURE 2:**
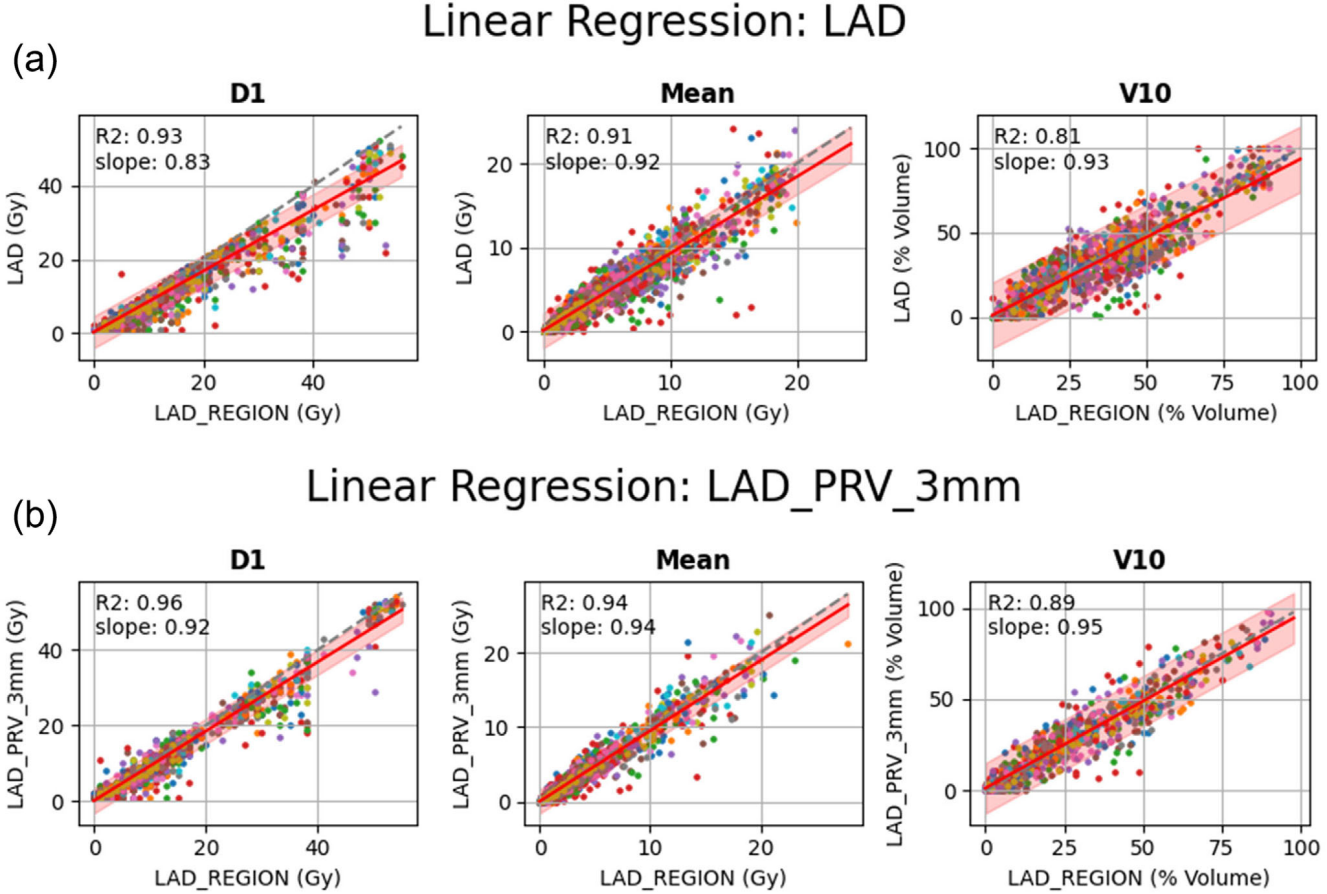
**Structure correlation**. For D1%, Mean, and V10Gy the total data points fit are *n* = 3116, *n* = 3034, *n* = 972 respectively. (A) Mean, volumetric and maximum point dose are shown for the LAD versus LAD_REGION. Mean absolute error is 0.5 Gy, 6.8%, and 1.1 Gy for mean, V10, and max dose respectively. (B) Same analysis is shown for the LAD_PRV_3 mm and LAD_REGION. Mean absolute error is 0.4 Gy, 5%, and 0.9 Gy respectively. The 95% confidence interval is shown by the red bands, and a perfect linear fit outlined by the dashed line.

We observed a strong relationship between dose metrics to the LAD and the LAD_REGION. Specifically, the maximum dose metric (D1%[Gy]) to the LAD_REGION closely tracked that of the LAD (*R* ^2^ = 0.93), with even stronger agreement when comparing to the LAD_PRV_3 mm (*R* ^2^ of 0.96) (Figure 2). This correlation remained strong for cut off doses up to 10 Gy (*R* ^2^ = 0.93) (Table 1).

**TABLE 1:** DVH metric correlation of LAD_REGION to LAD_PRV_3 mm with varying cut‐off doses.

Dose metric	*Δ* = 2 cGy	*Δ* = 2 Gy	*Δ* = 5 Gy	*Δ* = 10 Gy	*Δ* = 20 Gy
D1%	0.961	0.961	0.946	0.928	0.848
D2%	0.963	0.962	0.949	0.928	0.872
D5%	0.962	0.959	0.944	0.918	0.885
D10%	0.957	0.954	0.936	0.905	0.916
D15%	0.947	0.942	0.934	0.905	0.872
D20%	0.926	0.922	0.905	0.860	0.828
D25%	0.898	0.892	0.878	0.823	0.755
D30%	0.859	0.858	0.828	0.742	0.663
D35%	0.808	0.813	0.773	0.649	0.514
D40%	0.777	0.762	0.699	0.495	0.438
D45%	0.702	0.674	0.575	0.286	0.017
D50%	0.566	0.523	0.356	0.079	0.997
V5Gy	0.865	0.847	0.835	0.822	0.800
V10Gy	0.889	0.870	0.841	0.820	0.786
V15Gy	0.818	0.739	0.637	0.507	0.429
V20Gy	0.651	0.627	0.598	0.541	0.436
V25Gy	0.802	0.777	0.745	0.662	0.387
V30Gy	0.829	0.767	0.686	0.476	0.376
V35Gy	0.793	0.705	0.569	0.457	0.360
V40Gy	0.727	0.600	0.537	0.505	0.308
V45Gy	0.697	0.597	0.566	0.381	0.344
V50Gy	0.637	0.553	0.583	0.519	N/A
Mean Gy	0.936	0.900	0.831	0.541	0.000

*Note*: Data points with dose to the LAD_PRV_3 mm below the indicated cut off dose (*Δ*) were removed from the linear regression dataset. The number of remaining data points were *n* = 3092, *n* = 2687, *n *= 1497, *n* = 786, *n* = 235 from a *Δ* = 0.2, 2, 5, 10, and 20 Gy respectively. Clinically, our goal is to push the 2–10 Gy isodose line out of the LAD which maintains a strong correlation with maximum dose.

Importantly, V10Gy[%] shows strong agreement between the LAD_REGION and both the LAD (*R* ^2^ = 0.81), and LAD_PRV_3 mm (*R* ^2^ = 0.89) (Figure 2). Notably, the radiobiological effect of SBRT differs from IMRT, making the direct application of V15Gy[%] unsuitable. However, a simple BED calculation using a normal tissue alpha beta ratio for the LAD yields a hypothetical equivalent dose metric of V10Gy. Since this is an estimation of LAD alpha/beta, a large range of dose metrics are reported in the supplementary figures, while the focus remains on V10Gy[%] (Figure S2–S5). Finally, to validate our methodology, histograms demonstrated a high degree of residual normality, and the LMM random effect variance of 0 indicated negligible variance attributed to patient‐specific clustering for D1%[Gy], V10Gy[%], and mean dose (Figures S2–S5). Bland‐Altman plots are included in Figure S6.

### 3.3 MHD assessment

**FIGURE 3:**
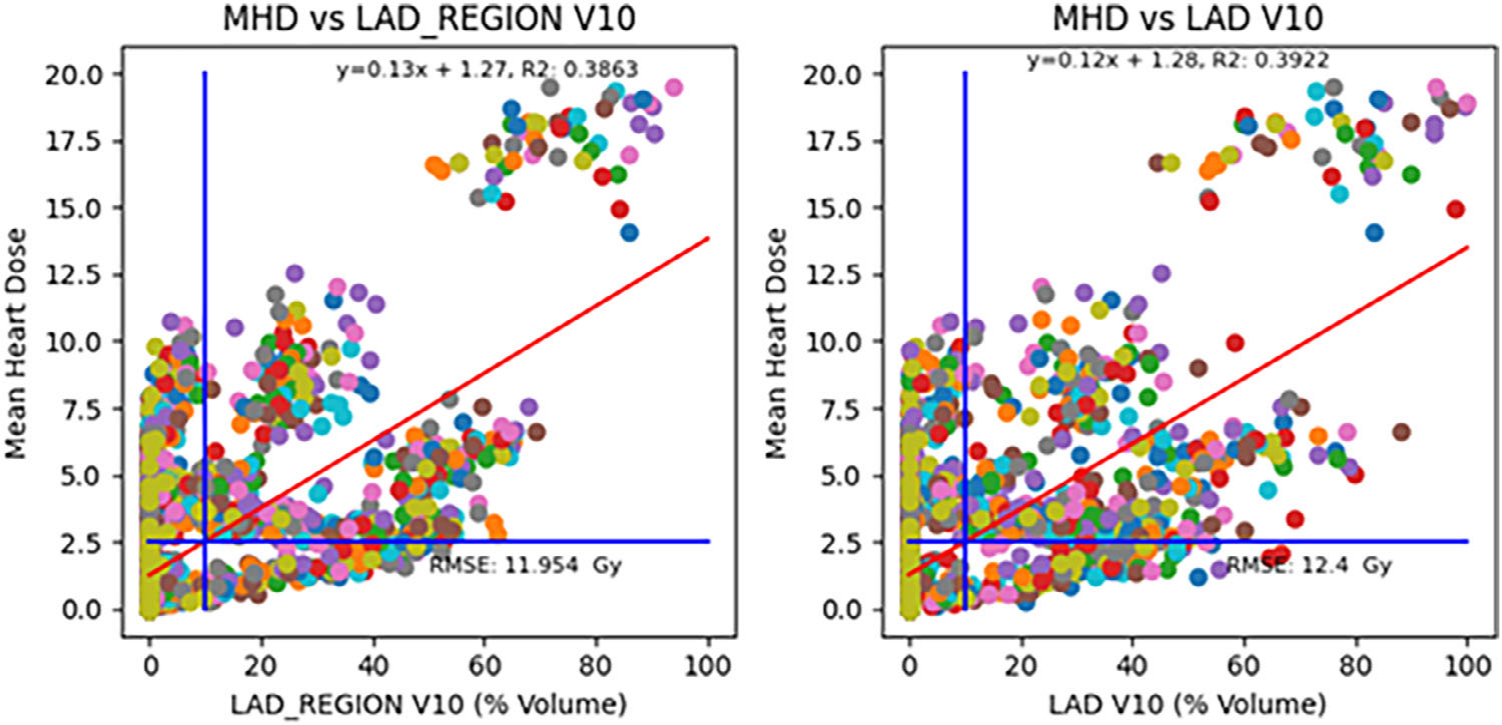
**MHD versus LAD**. MHD versus LAD_REGION V10Gy[%] (left) and the LAD V10Gy[%] (right). A low *R* ^2^ value of 0.386 and 0.392 (compared to the previously reported 0.545).

As expected, MHD showed similar poor correlation with V10Gy[%] of both LAD and LAD_REGION (*R* ^2^ ≈ 0.4) (Figure 3), highly consistent with prior reports in lung cancer patients where MHD has been shown to poorly correlate with doses to the LAD.^6^ Differences in slope compared to published studies likely reflect variations in prescription dose and dose fall‐off characteristics between SBRT and conventional IMRT/3D‐CRT.

## 4 DISCUSSION

Accurate estimation of LAD dose is a barrier to investigating the currently unknown clinical impact of LAD exposure in patients with early‐stage NSCLC treated with SBRT. On standard 4DCT scans, motion blurring and poor soft‐tissue contrast make the LAD difficult to visualize, limiting both clinical contouring and research efforts that rely on ground‐truth LAD structures. Advantageously, defining the region rather than the artery itself makes visualization on every CT slice nonessential, which may improve both manual review and auto segmentation model performance. Our results demonstrate that the LAD_REGION dose metrics strongly correlate with those of manually contoured LAD and LAD_PRV_3 mm in lung SBRT, which is particularly challenged by steep dose gradients. This finding suggests that the LAD_REGION, which is facilitated by AI and has been deployed clinically at our institution, may serve as a practical surrogate for LAD dosimetry. Clinically, our goal is to push the 2–10 Gy isodose line out of the LAD_REGION. Our findings suggest LAD doses at these levels strongly correlates with LAD_REGION dose (Table 1). As shown in the Bland‐Altman analysis (Figure S6), there is notable bias for D1% indicating an average over prediction of D1% of 1.3 Gy. This relationship is to be expected, given that the region generally encases the LAD. Despite the bias, the correlation remains strong indicating that using the D1% of the LAD_REGION is a strongly correlated and conservative surrogate to monitor LAD dose. The strength of the study is the use of this previously unreported AI model that was rigorously developed in collaboration with Google DeepMind and the validation of the predictive power of the LAD_REGION in lung SBRT dose distributions. No previous study has evaluated these dose parameter correlations between the AI model and the manually contoured LAD structure. This tool has the potential to enable large scale outcomes study to identify important dosimetric LAD goals in stage I NSCLC.

Two other open‐source LAD segmentation models intended for lung cancers were trialed on both DIBH and 4DCT scans prior to the commencement of this study, one which performed atlas segmentation, and a second which generated LAD contours using deep learning.^22^, ^23^ The trialed atlas segmentation model also reports a high mean dose correlation with the LAD with a RMSE of 2.7 Gy, but failed to report other dose metrics, IR and reported poor DSC of less than 0.1.^22^ In comparison, RMSE between LAD_PRV_3 mm and LAD_REGION mean dose using our model is < 1 Gy. From the deep learning open‐source model, mean absolute error in mean dose and maximum dose to the LAD were 1.39 Gy and 6.55 Gy respectively.^29^ Our model reports 0.4 Gy and 0.9 Gy respectively. While a full comparison of the three models is out of the scope of this study, a brief visualization of the performance of the various models is provided in Figure S7.

As mentioned, other published auto segmentation models report DSC scores ranging from 0.06 for the LAD and 0.6 for an LAD planning risk volume. Our DSC score of the LAD and the LAD_REGION was 0.175, with a standard deviation of 0.07. The DSC score of the LAD_PRV_3 mm and the LAD_REGION was improved to 0.55 with a standard deviation of 0.09. This geometric disagreement could cause significant differences in maximum dose reported, however if the LAD_REGION is specific enough to the region of the LAD, the correlation should remain strong. Additionally, the high‐volume disparity due to the difference in anatomical definition between the LAD_REGION and the LAD (mean of 32.9 cm3 vs. 2.9 cm3 respectively) makes DSC less suitable as a quality metric as it could never achieve an ideal value of 1. Despite this drawback, our model produces DSC scores comparable to previously reported models.

Our hybrid approach using clear anatomy and real lung SBRT dose distributions enabled robust dosimetric comparisons between manual LAD contours and an AI‐derived LAD_REGION surrogate, while avoiding the uncertainties inherent to LAD delineation on 4DCT. A trade‐off in this technique is the introduced uncertainty of patient specific LAD motion margins. Despite the simplification of the 1 cm safety margin around the LAD, it is preferable in this study as even manual contouring of the motion of the LAD on a 4DCT scan is inherently uncertain due to scan contrast.

This study has several limitations. First, variability in lung function and volume for stage I NSCLC patients are not considered in this approach. Ultimately, while stage I NSCLC lesions are typically small, a model trained on DIBH scans may behave unpredictably when applied to a 4DCT scan featuring a high‐density lung lesion. While this study focuses on the LAD_REGION and LAD dose correlation using clear DIBH scans, further evaluation of model robustness on the most challenging 4DCT scans (artifact, abnormal anatomy, and proximity of LAD to lung lesions) has shown promise and is currently under investigation.

Additionally, it is well known that acute breath hold elicits complex physiological responses that impact the cardiovascular system.^30^ However, in terms of heart morphology itself, little data is available. One recent study looking at real time cine MRI versus standard of care breath hold MRI compared metrics such as ejection fraction, end diastolic volume, end systolic volume, left ventricular mass, and wall thickness under both imaging regimes. They found no statistically significant difference in any of these metrics, indicating minimal change and supporting our approach.^31^ In our methodology, the rotation of the heart under breath hold was not accounted for, as we performed only three‐degrees‐of‐freedom rigid registration to simulate tumor position and treatment. Rigid registration presents two major drawbacks for this study. Significantly, rigid registration does not account for variations in overall heart size. Secondly, breath hold may change the relative position of the heart within the lungs. Our registration technique that optimized for the left anterior portion of the heart minimized these effects. Additionally, the quality of the registrations was dependent on standardized heart contouring of the superior edge, which we define as the bifurcation of the pulmonary trunk. More advanced registration techniques, such as deformable registration, were not pursued due to its potential to significantly distort dose gradients or contour geometry.

## 5 CONCLUSION

Our study indicates that the AI generated LAD_REGION structure provides a practical surrogate for LAD dose estimation in lung SBRT dose gradients. Leveraging an AI‐based model, LAD_REGION has the potential to enable scalable and reproducible assessment of clinically significant LAD dose across large patient cohorts.

## AUTHOR CONTRIBUTIONS

Grace Chang contributed to data curation, formal analysis, investigation, methodology development, validation, and visualization. She also played a key role in drafting the original manuscript and participated in the review and editing process. Nathan Y. Yu contributed to investigation and methodology, and was responsible for project administration, resource provision, supervision, validation, and visualization. He also contributed to the manuscript through review and editing. Randa Tao was involved in data curation, investigation, and methodology, and supported project administration and supervision, as well as manuscript review and editing. Yi Rong contributed to investigation, methodology, project administration, and resource provision, and was responsible for software development, supervision, and visualization. He also contributed to both the original draft and the review and editing of the manuscript. Libing Zhu participated in investigation, methodology, and visualization, and contributed to the manuscript through review and editing. Quan Chen contributed to data curation, formal analysis, investigation, methodology, project administration, and resource provision. He also led software development, supervision, validation, and visualization efforts, and contributed to both the original draft and the review and editing of the manuscript.

## CONFLICT OF INTEREST STATEMENT

Yi Rong is current Editor in Chief for JACMP but will have no role in the review of this manuscript.

## ETHICAL APPROVAL

This study was conducted in accordance with institutional policy and federal regulations governing human subjects research. The protocol received approval from the Mayo Clinic Institutional Review Board (IRB #25‑001326, approval date: April 8, 2025) and was reviewed under expedited procedures (45 CFR 46.110; 21 CFR 56.110). The study was determined to represent minimal‑risk research under Expedited Categories 1(b) and 5. The IRB granted a waiver of informed consent and waiver of HIPAA authorization due to the retrospective design and minimal risk to participants, with approval for the inclusion of 300 subjects.

## Supporting information



Supporting Information
