# Supplementary material for: AI‐surrogate structure and dose correlation for left anterior descending artery in lung stereotactic body radiotherapy
Source: J Appl Clin Med Phys. 2026 Jul 8;27(7):e70703. doi: 10.1002/acm2.70703 (PMC13344228; doi:10.1002/acm2.70703)
Supplement: Supplementary file 1 — Supporting Information [file ACM2-27-e70703-s001.docx]

**Fig S1**. Inclusion Ratio distributions for LAD and LAD_PRV_3mm


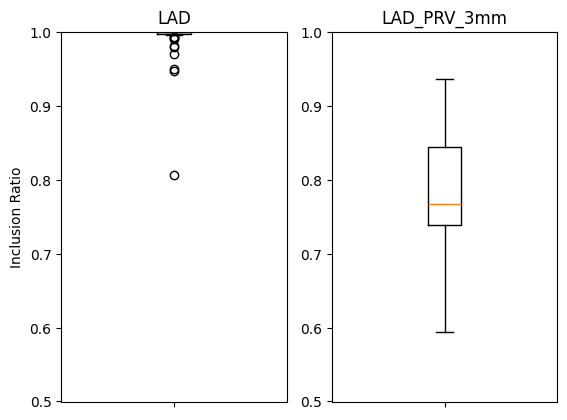


Inclusion ratio is defined by the percentage of the LAD or LAD_PRV_3mm inside of the LAD region

**Fig S2.** Histogram of residuals for LAD vs LAD_REGION


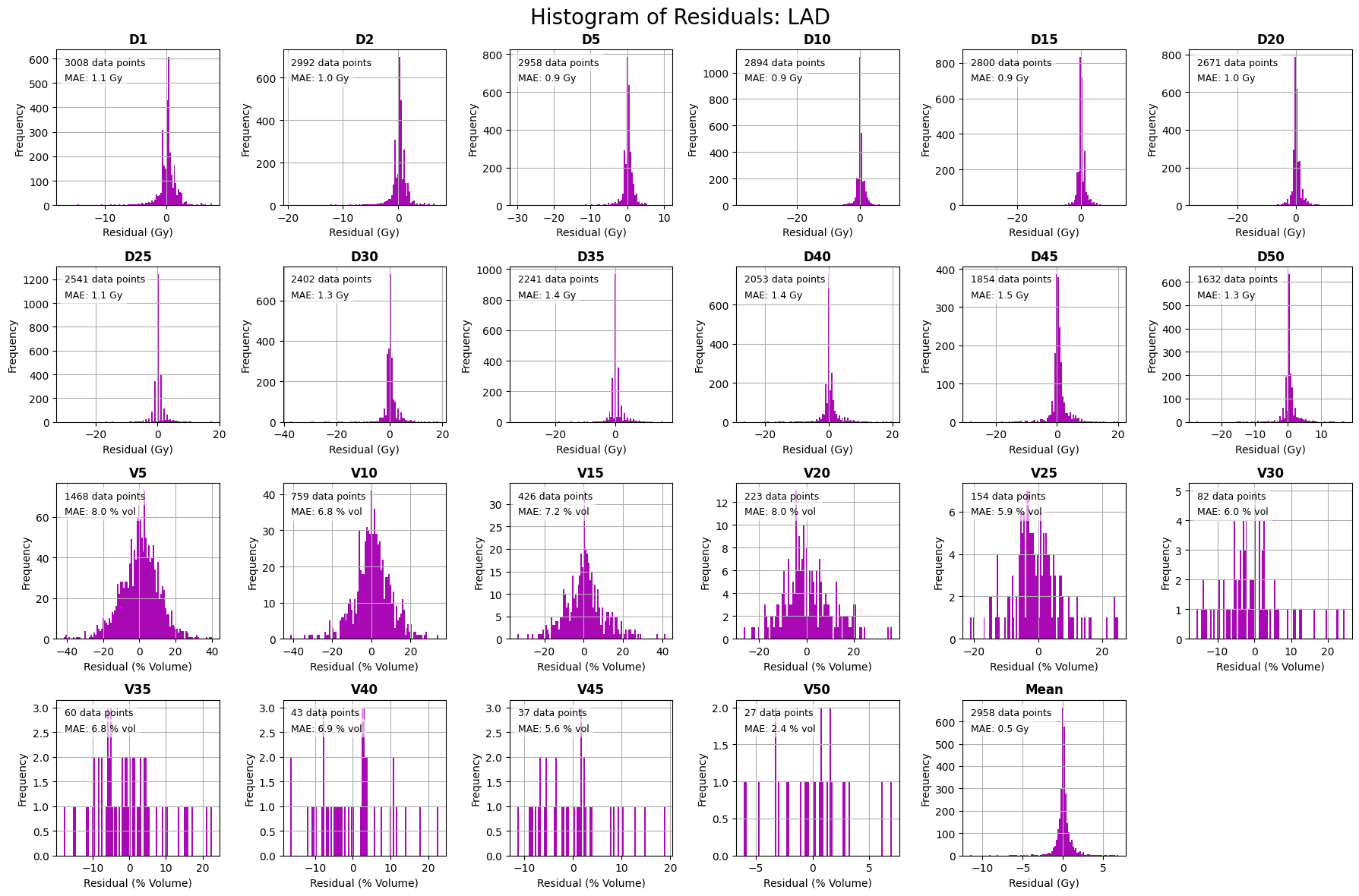


**Figure S3.** Linear regression for LAD vs LAD REGION


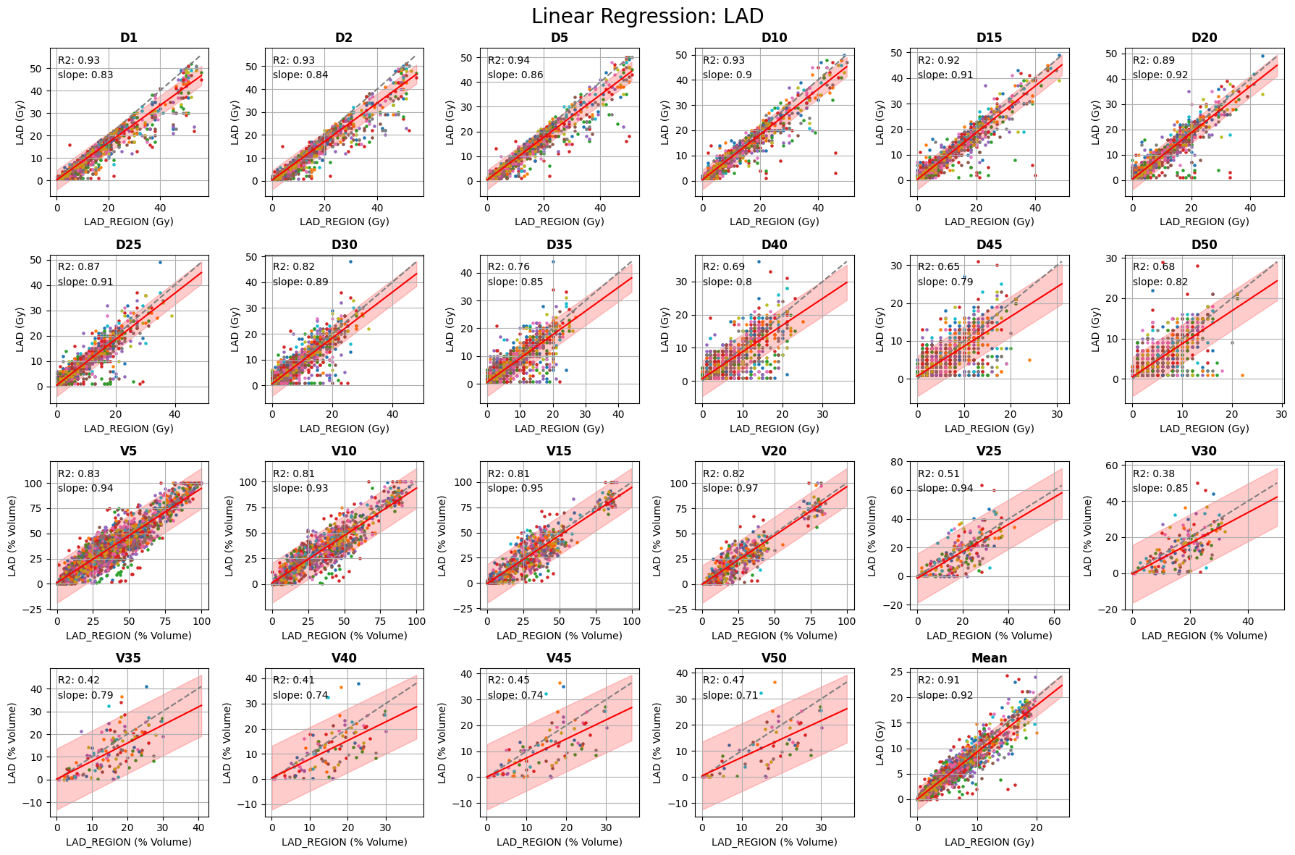


**Figure S4.** Histogram of residuals for LAD_PRV_3mm vs LAD_REGION


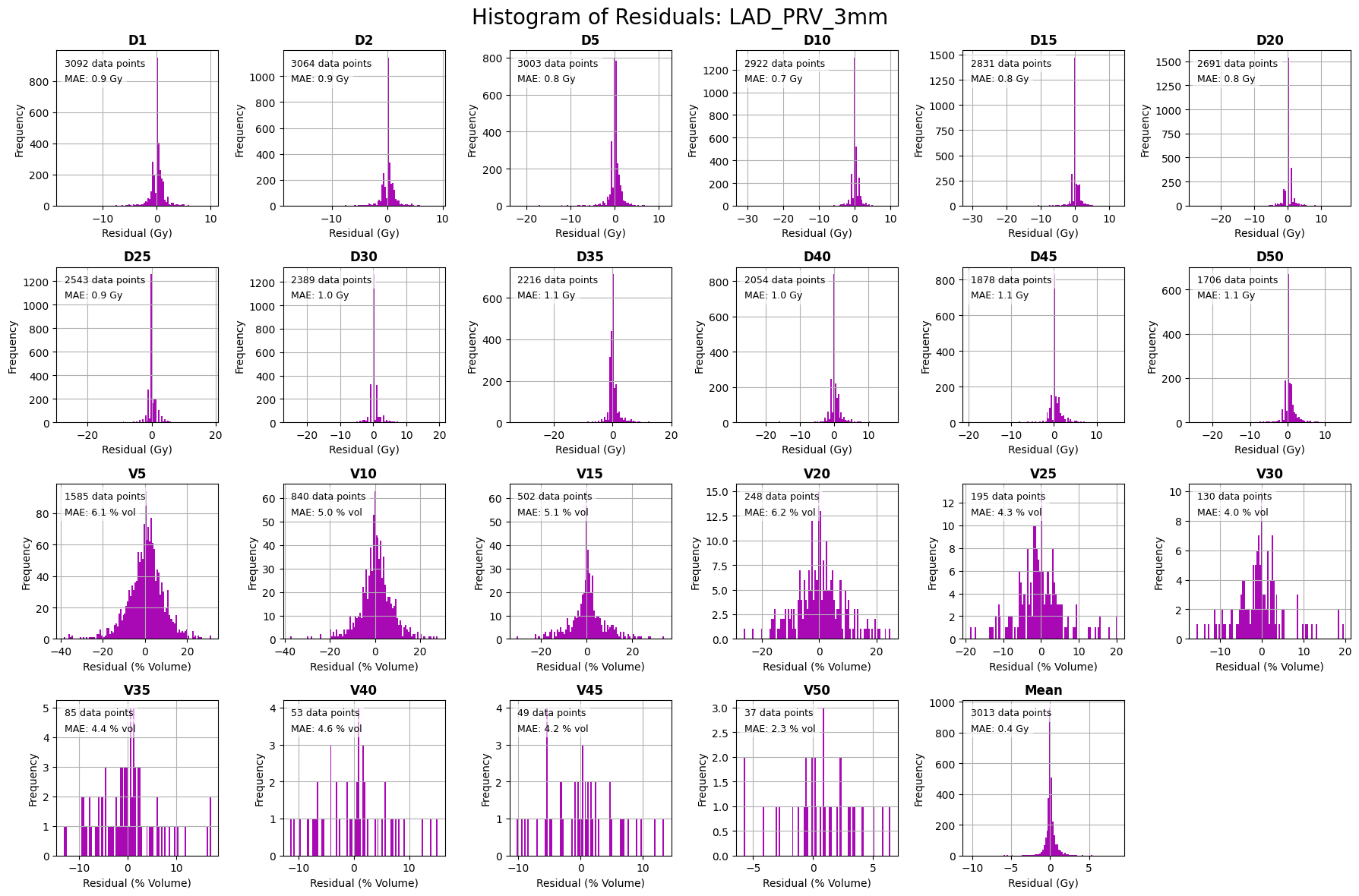


**Figure S5.** Linear Regression for LAD_PRV_3mm vs LAD REGION


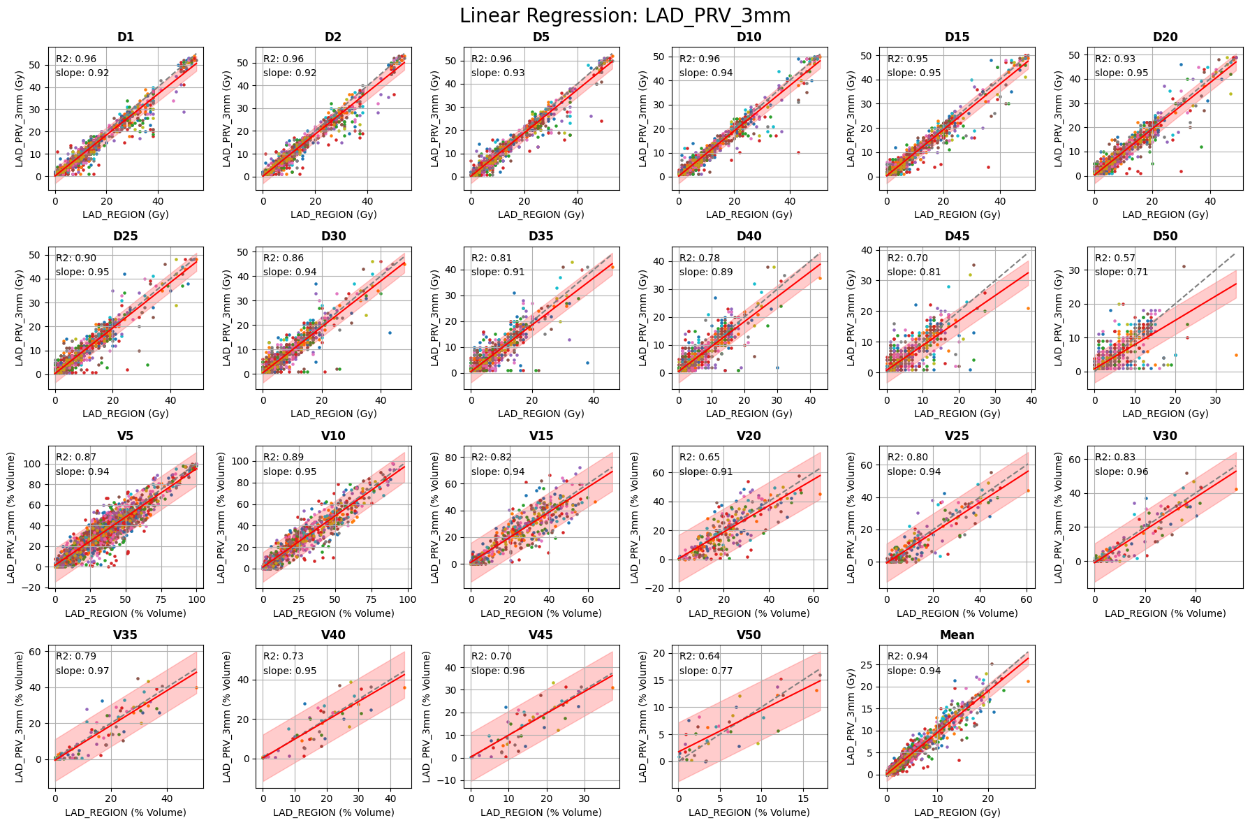


**Figure S6.** Bland Altman Plots


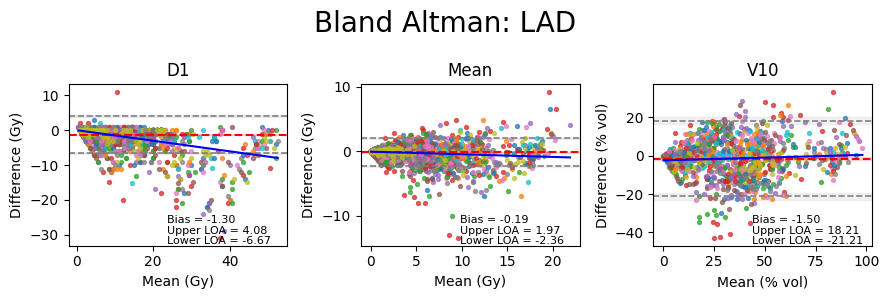

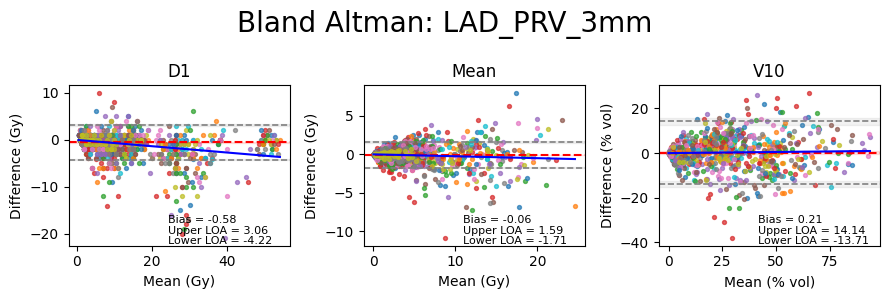


Red dashed line is 0 difference, blue is bias, and gray dashed bands are the upper and lower LOA.

**Figure S7.** Various open source models for LAD delineation on one patient


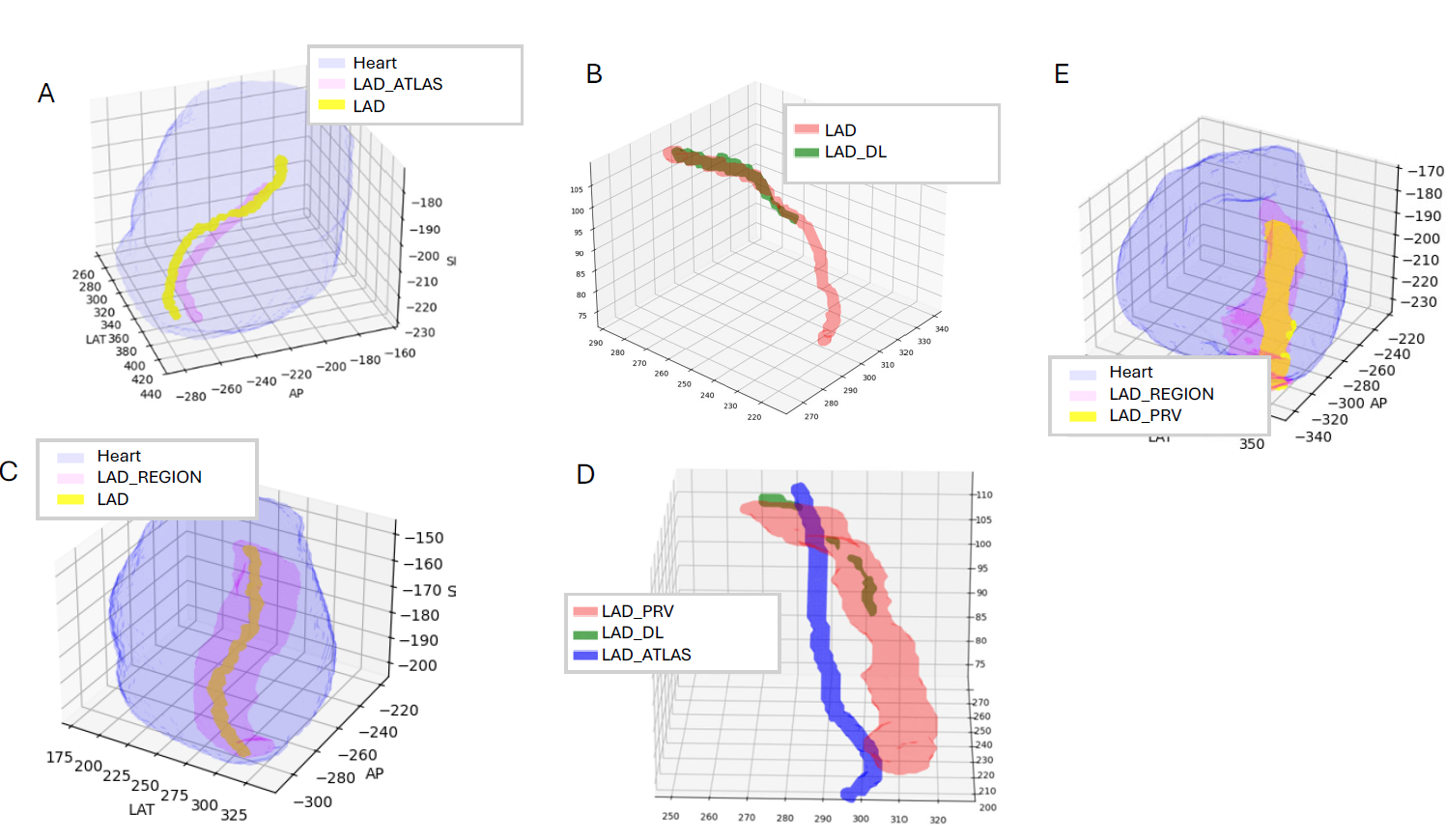


Panels A-C demonstrate various auto segmentation models on a DIBH scan including atlas based (A), deep learning (B) and our LAD_REGION model (C). Panels D-E demonstrate the same models tested on a 4DCT scan.
